# Detecting Cancer Survival Related Gene Markers Based on Rectified Factor Network

**DOI:** 10.3389/fbioe.2020.00349

**Published:** 2020-04-23

**Authors:** Lingtao Su, Guixia Liu, Juexin Wang, Jianjiong Gao, Dong Xu

**Affiliations:** ^1^Department of Electrical Engineering and Computer Science, University of Missouri, Columbia, MO, United States; ^2^Department of Computer Science and Technology, Jilin University, Changchun, China; ^3^Memorial Sloan Kettering Cancer Center, New York, NY, United States

**Keywords:** rectified factor network, biclustering, survival analysis, biomarker, variational inference

## Abstract

Detecting gene sets that serve as biomarkers for differentiating patient survival groups may help diagnose diseases robustly and develop multi-gene targeted therapies. However, due to the exponential growth of search space imposed by gene combinations, the performance of existing methods is still far from satisfactory. In this study, we developed a new method called BISG (BIclustering based Survival-related Gene sets detection) based on a rectified factor network (RFN) model, which allows efficiently biclustering gene subsets. By correlating genes in each significant bicluster with patient survival outcomes using a log-rank test and multi-sampling strategy, multiple survival-related gene sets can be detected. We applied BISG on three different cancer types, and the resulting gene sets were tested as biomarkers for survival analyses. Secondly, we systematically analyzed 12 different cancer datasets. Our analysis shows that the genes in all the survival-related gene sets are mainly from five gene families: microRNA protein coding host genes, zinc fingers C2H2-type, solute carriers, CD (cluster of differentiation) molecules, and ankyrin repeat domain containing genes. Moreover, we found that they are mainly enriched in heme metabolism, apoptosis, hypoxia and inflammatory response-related pathways. We compared BISG with two other methods, GSAS and IPSOV. Results show that BISG can better differentiate patient survival groups in different datasets. The identified biomarkers suggested by our study provide useful hypotheses for further investigation. BISG is publicly available with open source at https://github.com/LingtaoSu/BISG.

## Introduction

Identifying biomarker genes for survival risk prediction allows earlier detection of mortality risk and design of individualized therapy (Wang and Liu, 2018). Due to the exponential growth of search space imposed by the combination explosion of genes, most proposed survival prediction models mainly focus on a single gene. However, the genes perform their functions as groups rather than individually. Identifying robust gene sets that can consistently predict a patient's survival outcome has become a main challenge in the field.

In gene expression experiments, functionally related genes often exhibit a similar pattern in only a subset of samples or under specific experimental conditions (Padilha and Campello, 2017). This problem can be solved by biclustering, which can be used to detect latent row and column groups of different response patterns (Zhang et al., 2017; Saelens et al., 2018). By combining patient survival information, whether the resulting subset of genes are related to patient survival can be tested. Sparse coding has demonstrated its advantage in biclustering gene expression data (Hochreiter et al., 2010). Using sparse representations, the biclustering model tends to have a smaller number of row and column groups since a large amount of variation is already explained by these observed covariates (Blei et al., 2017). In fact, sparse coding has been well-developed in deep learning obtained by rectified linear units (ReLU) (Xu et al., 2016) and dropout (Srivastava et al., 2014). Recently, the rectified factor network (RFN) model (Clevert et al., 2015) was introduced, which aims at finding a sparse, non-negative representation of the input, and extracting the covariance structure of the data. The RFN model uses the posterior regularization method (Ganchev et al., 2010), which separates model characteristics from data dependent characteristics and restricts the posterior means to be non-negative. As computing posterior is very time consuming, variational inference is utilized in RFN model, which approximates probability densities through optimization. Furthermore, by utilizing the projected Newton and projected gradient update strategies during optimization, RFN can efficiently carry out biclustering with high accuracy.

In this study, we adapted RFN for biclustering analysis of integrated mutation and gene expression datasets from the same sets of samples, and developed a new method called BISG (BIclustering based **S**urvival-related **G**ene sets detection). As in Hochreiter et al. (2010), a bicluster is defined as a pair of a row (gene) set and a column (sample) set for which the rows are similar to each other on the selected columns and vice versa. The motivation for developing BISG is to predict such biclusters using gene expression data and associate these biclusters with diseases and disease subtypes. BISG is a rectified factor analysis model, which extracts the covariance structure of the input data and enforces the posterior has to be non-negative and normalized. Non-negative constraints lead to sparse and non-linear codes, while normalization constraints scale the signal part of each hidden unit. For computing the posterior, a family of variational distribution *Q* of allowed posterior distributions is introduced. In this way, we transform the biclustering problem into an optimization problem, which is optimized by a generalized alternating minimization algorithm (Gunawardana and Byrne, 2005). To speed up computation in the generalized expectation maximization algorithm, we perform a gradient step in both E-step and M-step with fast GPU implementations. We correlate genes in each significant bicluster with patient survival outcomes using a log-rank test and multi-sampling strategy, and only keep the gene sets that can differentiate sample groups by their significantly different survival curves in training and validation datasets. The identified biomarkers suggested by our study can be used as hypotheses for further investigation in improving cancer patient survival.

## Materials and Methods

### Methods Overview

**Figure 1:**
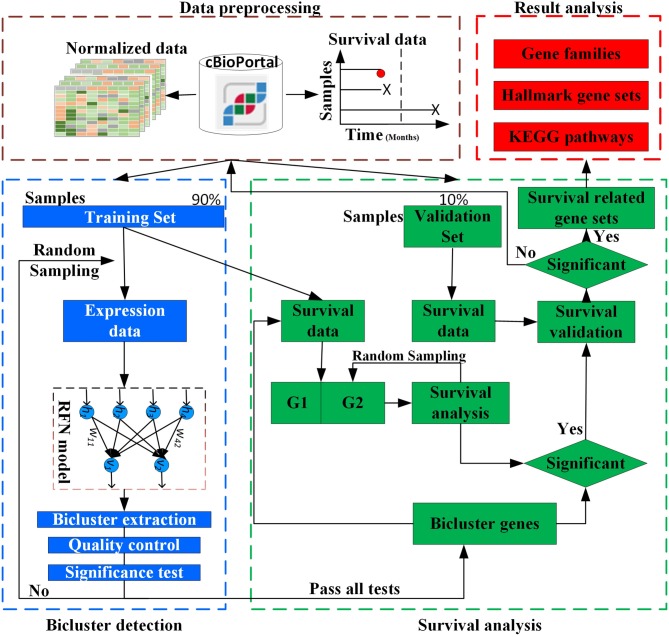
Overview of BISG.

The overall design of BISG is shown in Figure 1. BISG mainly comprises of four parts: (1) data preprocessing, (2) bicluster detection, (3) survival analysis, and (4) result analysis. BISG takes RNAseq data, single nucleotide polymorphisms (SNP) data and sample survival data as input. In the data preprocessing, only genes having at least one SNP mutation and samples with survival information are kept. The expression data are normalized to a range between 0 and 1. Each time 90% of the samples are iteratively used as a training set to detect significant biclusters, and the remaining 10% are then used as a validation set. For bicluster detection, a multi-sampling strategy is applied. Each time we randomly select expression data of 100 different samples from the training set to detect significant biclusters using the RFN model, bicluster extraction, quality control and significance test methods. Biclusters passing all these tests are then used for survival analysis. Based on the genes in each bicluster, BISG separates samples (patients) in the training set into two groups G1 (with over 80% bicluster genes significantly up-regulated) and G2 (with all bicluster genes express normally). The survival curves of the two groups are statistically tested by a log-rank test. A multi-sampling strategy is also used in this test, i.e., each time we randomly select the same number of samples from G2 as in G1 (or from G1 as in G2, depending on which one has more samples). If a bicluster gene set can differentiate sample groups by their significantly different survival curves in 80% samplings in the training set, we then validate whether the bicluster genes can separate patients in the validation set into two different survival groups. We random sample 1,000, 5,000, and 10,000 times respectively, and after all iterations only commonly occurred significant bicluster gene sets that can well separate patients in the validation set into different survival groups are selected as biomarkers. In the result analysis, we conduct an independent test of biomarkers with new datasets from GEO (Gene Expression Omnibus) database, and do KEGG and hallmark gene sets enrichment analysis, and also identify common gene families of all the biomarker genes.

### Data Preprocessing

Table 1 summarizes the data of the 12 cancer types used in training and validation of BISG. We downloaded their RNAseq median Z-score datasets, SNP mutation datasets and clinical datasets from the cBioPortal database (Cerami et al., 2012; Gao et al., 2013). Based on the median Z-score value we normalized each gene expression values to a range between 0 and 1 (0 means no change, 1 means highly up-regulated).

**Table 1:** Cancer data used for training and validating biomarkers.

**ID**	**Cancer type**	**Gene number**	**SNP number**	**Sample number**
1	Brain lower grade glioma	2,511	3,141	282
2	Colorectal adenocarcinoma	10,680	23,982	222
3	Glioblastoma	4,148	5,974	130
4	Head and neck squamous cell carcinoma	11,767	27,742	500
5	Kidney renal clear cell carcinoma	6,572	9,923	435
6	Lung adenocarcinoma	8,180	16,625	221
7	Ovarian serous cystadenocarcinoma	3,641	4,573	183
8	Pancreatic adenocarcinoma	6,101	9,415	150
9	Papillary thyroid carcinoma	1,320	1,437	313
10	Prostate adenocarcinoma	7,673	12,658	496
11	Thyroid carcinoma	1,656	1,835	395
12	Breast Invasive Carcinoma	7,079	11,089	448

After the biomarkers were predicted, we utilized three microarray datasets GSE16011 (Gravendeel et al., 2009), GSE3494 (Palazon et al., 2017), and GSE11969 (Takeuchi et al., 2006), as well as their corresponding sample survival information from the GEO as independent test datasets to confirm these biomarkers detected in gliomas, breast cancer and lung adenocarcinoma, respectively. Two datasets, GSE1456 (Pawitan et al., 2005), which was used by GSAS (Varn et al., 2015) but not BISG, and GSE32062 (Yoshihara et al., 2012), which was used by IPSOV (Shen et al., 2019) but not BISG, were used to compared the classification performance of gene sets detected by BISG, GSAS, and IPSOV. Another dataset GSE3494 (new data for BISG and GSAS) was used to test whether the core gene set detected by GSAS and the top-ranked gene set identified by BISG with breast cancer datasets from cBioPortal database can differentiate samples in GSE3494 into different survival groups. These datasets were normalized the same as in the cBioPortal database, and the datasets were shown in Table 2.

**Table 2:** Independent test datasets used for confirming predicted biomarkers and for comparison.

**ID**	**Cancer name**	**Gene number**	**Sample number**
GSE3494	Breast cancer	4,883	236
GSE11969	Lung Adenocarcinoma	5,273	149
GSE16011	Gliomas	2,061	264
GSE1456	Breast cancer	14,204	159
GSE32062	Ovarian cancer	19,592	260

### Bicluster Detection

Given a normalized gene expression matrix, *V* = (*X, Y*), with a set of rows *X* = {*x*_1_, …, *x*_*N*_}, a set of columns *Y* = {*y*_1_, …*y*_*M*_}, and the element *v*_*ij*_ ∈ *V* represents the expression value of gene *i* in sample *j*. A bicluster *B* = (*I, J*) is a *n* × *m* submatrix of *V*, where *I* = (*i*_1_, …*i*_*n*_) ⊂ *X* is a subset of genes and *J* = (*j*_1_, …*j*_*m*_)⊂*Y* is a subset of samples. The biclustering aims to identify a set of biclusters *B* = {*B*_1_, …*B*_*s*_} such that each bicluster *B*_*k*_ = (*I*_*k*_, *J*_*k*_) satisfies specific homogeneity criteria. The RFN model is a single or stacked factor analysis model as in Equation (1), which extracts the covariance structure of the data.

(1) $$\begin{array}{l} V=Wh+\varepsilon \end{array}$$

where *V* = {*V*_1_, …*V*_*N*_) is the input data (visible units), *h* ~ *N*(0, *I*) is the hidden unit (where *N* is a normal distribution), *W* is the weight matrix, ε ~ ℕ(0, Υ) is the noise error vector, and Υ is the noise covariance matrix. The parameters of the model are *W* and Υ. If *h* is given, then only the noise ε is a random variable and we have *V*|*h* ~ *N*(*Wh*, Υ).

Let *E* denote the expectation of the data including the prior distribution of the factors and the noise distribution. We can get *E*(*VV*^*T*^) = *WW*^*T*^ + Υ. The marginal distribution for *V* is *V* ~ *N*(0, *WW*^*T*^ + Υ). The log-likelihood of the input data is given in Equation (2).

(2) $$\begin{array}{l} \log\prod_{i=1}^{n}p(V_{i})=-\frac{nm}{2}\log(2\pi )-\frac{n}{2}\log|WW^{T}+\Upsilon |\\ \;-\frac{1}{2}\sum_{i=1}^{n}V_{i}^{T}{\left(WW^{T}+\Upsilon \right)}^{-1}V_{i} \end{array}$$

For the mean-centered input vector *V*, the posterior *p*(*h*_*i*_|*V*_*i*_) is Gaussian with the mean vector (_*u*_*p*_)*i*_ and covariance matrix *K*_*pp*_ as in Equation (3):

(3) $$\begin{array}{l} {\left(u_{p}\right)}_{i}={\left(I+W^{T}\Upsilon^{-1}W\right)}^{-1}W^{T}\Upsilon^{-1}V_{i},K_{pp}\\ \;={\left(I+W^{T}\Upsilon^{-1}W\right)}^{-1} \end{array}$$

To maximize the likelihood, we introduce a variational distribution *Q*, and the objective function *F* of our model is shown in Equation (4):

(4) $$\begin{array}{l} \text{ 𝔽}=\frac{1}{n}\overset{n}{\underset{i=1}{\sum }}\log p\left(V_{i}\right)-\frac{1}{n}\overset{n}{\underset{i=1}{\sum }}D_{KL}\left(Q\left(h_{i}|V_{i}\right)||p\left(h_{i}|V_{i}\right)\right)\\ =\frac{1}{n}\overset{n}{\underset{i=1}{\sum }}\int Q\left(h_{i}|V_{i}\right)\log p\left(V_{i}|h_{i}\right)dh_{i}-\frac{1}{n}\overset{n}{\underset{i=1}{\sum }}D_{KL}\left(Q\left(h_{i}|V_{i}\right)||p\left(h_{i}\right)\right) \end{array}$$

where *Q* is a variational distribution for the approximate of the posterior *p*(*h*_*i*_|*V*_*i*_). We constrain *Q* to the family of rectified and normalized Gaussian distributions. *D*_*KL*_ > 0 is the KL distance. 𝔽 is the objective of the EM algorithm. The E-step maximizes 𝔽 with respect to *Q*; therefore, the E-step minimizes *D*_*KL*_(*Q*(*h*_*i*_|*V*_*i*_)||*p*(*h*_*i*_|*V*_*i*_)). The M-step maximizes 𝔽 respect to the parameters (*W*, Υ); therefore, the M-step maximizes ∫*Q*(*h*_*i*_|*V*_*i*_)log*p*(*V*_*i*_|*h*_*i*_)*dh*_*i*_. Considering the quadratic problem of the posterior regularization method, to speed up the computation using fast GPU implementations, we perform a gradient step in both E- and M-steps. In the E-step, we use the projected Newton method as in Equation (5).

(5) $$\begin{array}{l} \underset{\mu_{i}}{\min}\frac{1}{n}\overset{n}{\underset{i=1}{\sum }}{\left(\mu_{i}-{\left(\mu_{p}\right)}_{i}\right)}^{T}\left(\mu_{i}-{\left(\mu_{p}\right)}_{i}\right),\;s.t.\;\mu_{i}\geq 0,\\ \frac{1}{n}\overset{n}{\underset{i=1}{\sum }}\mu_{ij}^{2}=1 \end{array}$$

In Equation (5), with $\frac{1}{n}\overset{n}{\underset{i=1}{\sum }}\mu_{ij}^{2}=1,\mu_{i}\geq 0$ we constrain the variational distributions to the family of normal distributions with non-negative mean components, and can avoid the explaining away problem as shown in Clevert et al. (2015).

In M-step, we decrease the expected reconstruction error, as in Equation (6).

(6) $$\begin{array}{l} \varepsilon =\frac{1}{2}\left(m\log\left(2\pi \right)\right)+\log|\Upsilon |+Tr\left(\Upsilon^{-1}C\right)-2Tr\left(\Upsilon^{-1}W^{T}\right)\\ +Tr\left(W^{T}\Upsilon^{-1}WZ\right) \end{array}$$

Where $P=\frac{1}{n}\overset{n}{\underset{i=1}{\sum }}{V_{i}\mu }_{i}^{T}$, $Z=\frac{1}{n}\overset{n}{\underset{i=1}{\sum }}{V_{i}\mu }_{i}^{T}+K_{pp}$ and $C=\frac{1}{n}\overset{n}{\underset{i=1}{\sum }}{V_{i}V}_{i}^{T}$. In combination, we get the updates for E-step: $E_{Q}\left(h_{i}\right)=\mu_{i},E_{Q}\left(h_{i}h_{i}^{T}\right)=\mu_{i}\mu_{i}^{T}+K_{pp}$ and M-step: *W*^*new*^ = *PZ*^−1^, Υ^*new*^ = *C*−*PW*^*T*^−*WP*^*T*^+*WZW*^*T*^.

**Figure 2:**
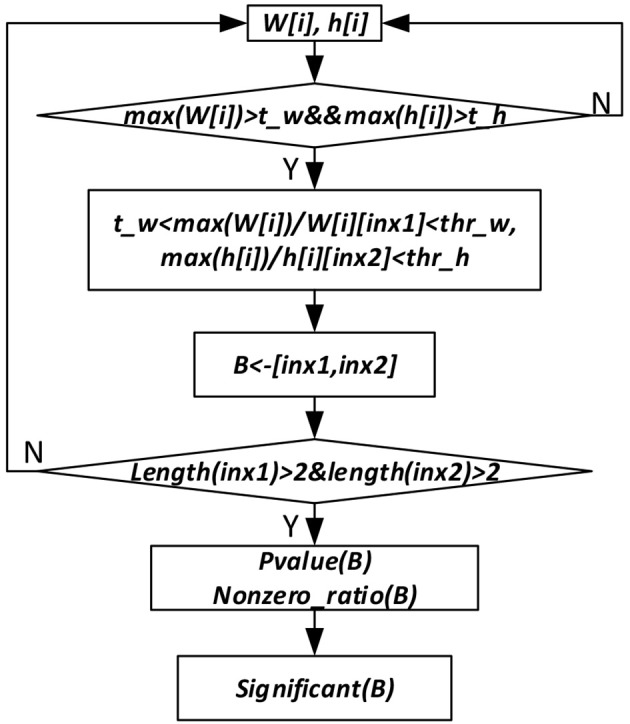
Significant bicluster extraction process. *W*[*i*] and *h*[*i*] are the gene and sample membership vectors. *max* (*W*[*i*]) and *max*(*h*[*i*]) are maximum values of *W*[*i*] and *h*[*i*], respectively. *t_w, t_h, thr_w*, and *thr_h* are threshold values used to filter bicluster membership genes and samples. *B* represents bicluster. *P-value* (*B*) is *p*-value of a bicluster *B*. *Nonzero_ratio* (*B*) is used for bicluster quality control, which is calculated as the ratio of non-zero elements in a bicluster.

To get the sparse, non-negative and non-linear of the input representations, and also to model the covariance structure of the input, we choose the maximum likelihood factor analysis as the model and apply the posterior regularization method (Ganchev et al., 2010). To enforce sparse codes, a Laplace prior on the weight matrix and dropout strategy are used. To further enforce sparseness of the sample and gene membership vectors, we propose a new bicluster extraction strategy as shown in Figure 2. For each gene and sample membership vectors, firstly, we get their maximum values, and then for each non-zero element, we get the ratio between the maximum value and the element. If the ratio fulfills the threshold value and at least two genes and two samples are included, then the bicluster is filtered for quality and significance test. For each bicluster passing the quality measure, a *p*-value (Equation 7) is calculated and the Bonferroni correction is used to control the overall type I error.

(7) $$\begin{array}{l} \mathrm{Pr}\left(B\left(m,n,q\right)\geq k\right)\geq \mathrm{Pr}\left(B\left(m,n,q\right)\geq mnq\left(1+\frac{k}{mnq}-1\right)\right) \end{array}$$

According to Koyuturk et al. (2004), if there is no association in a data matrix, each element can be assumed to an outcome of an independent Bernoulli trial with success probability *q*. Given a normalized gene expression matrix *V* with *M* rows, *N* columns and *K* none zero elements, we look for a subset of rows and columns such that a bicluster induced by these rows and columns is dense enough to be considered statistically significant. Assume that *Pr*(*V*(*i, j*) ≠ 0) = *q*, where *q* can be estimated by the density of the matrix, i.e., *q* = *K*/*MN*. For an arbitrary bicluster, with *m* rows and *n* columns, we assume that the number of non-zero elements is *k*. Then *k*follows a binormal distribution. The *p*-value of statistical significance test for an *m* × *n* bicluster is given in Equation (7). By using Chernoff's bound (Theodosopoulos, 2007), we get:

(8) $$\begin{array}{l} \mathrm{Pr}\left(k\geq mnp\left(1+\delta \right)\right)\leq e^{-mnp\delta^{2}/3} \end{array}$$

where δ > 0. Assume that the probability of observing *k* non-zero elements in the bicluster is less than P^*^, then by Equation (8), the bicluster is significant if *k* ≥ *mnp*(1 + δ), and $\delta \geq \sqrt{3\left(-\ln\;P^{*}\right)/mnp}$. In summary, according to Koyuturk et al. (2004) the bicluster is statistically significant if:

(9) $$\begin{array}{l} C\left(m,n,k\right)=k-mnp-\sqrt{3\left(-\ln\;P^{*}\right)/mnp}\geq 0 \end{array}$$

For each bicluster identified, the Bonferroni correction is used to control the overall type I error. The level of significance is set at $\frac{0.05}{b}$, where *b* is the number of biclusters identified. Besides, we use the none zero ratio in a bicluster to do quality control of the biclustering results. As defined above, the higher the k value, the better the quality of the identified bicluster.

### Survival Analysis

We use Kaplan-Meier plots (Goel et al., 2010) to visualize survival curves and with a log-rank test (Singh and Mukhopadhyay, 2011) to compare the survival curves of patients with and without changed expression of the bicluster gene sets. The survival probability, also known as the survivor function S(t), is the probability that an individual survives from the time origin (e.g., diagnosis of cancer) to a specified future time t. The survival probability at time t_i_, S(t_i_) is calculated as below:

(10) $$\begin{array}{l} S\left(t_{i}\right)=S\left(t_{i-1}\right)\left(1-d_{i}/n_{i}\right) \end{array}$$

where *S*(*t*_*i*−1_) is the probability of being alive at *t*_*i*−1_. *n*_*i*_ is the number of patients alive just before *t*_*i*_. *d*_*i*_ is the number of events at *t*_*i*_. *t*_0_ = 0 and *S*(0) = 1.

Considering genes in each significant bicluster, both samples in the training set and validation set can be divided into two groups G1 (with over 80% bicluster genes significantly changed) and G2 (with bicluster genes express normally). To test the survival difference of samples in G1 and G2, a multi-sampling strategy is utilized, each time the same number of samples are selected. The survival curves of the two selected sample groups can be compared statistically by testing the null hypothesis i.e., there is no difference regarding survival among two groups. This null hypothesis is statistically tested by a log-rank test. In the log-rank test, we calculate the expected number of events in each group, i.e., E1 and E2, while O1 and O2 are the total number of observed events in each group, respectively. The test statistic is:

(11) $$\begin{array}{l} Log-rank\;test={\left(O_{1}-E_{1}\right)}^{2}/E_{1}+{\left(O_{2}-E_{2}\right)}^{2}/E_{2} \end{array}$$

The test statistic and the significance can be drawn by comparing the calculated value with the critical value (using the chi-square table). To guarantee that the bicluster genes are more likely survival-related, for each significant bicluster, considering samples in the training set, we repeat the log-rank test 100 times. If the genes in the bicluster can separate patient groups in more than 80% sampling times, then we use the validation datasets to test whether they can also separate them into two different survival groups. Only bicluster gene sets passing all these significance tests are filtered out as the final biomarkers. We also confirm some biomarkers with independent datasets from the GEO database. In this study, the log-rank test and survival analysis are conducted based on functions in the *lifelines* python package.

## Results

### Biomarker Gene Sets in Brain Lower Grade Glioma, Lung Adenocarcinoma, and Breast Invasive Carcinoma

**Figure 3:**
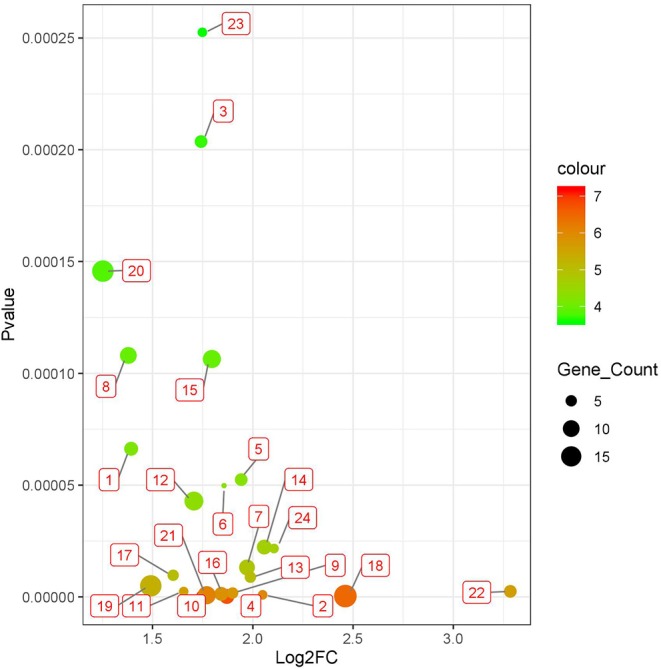
Twenty four significant survival-related gene sets detected in brain lower grade glioma with datasets from the cBioPortal database (Table 1). The corresponding genes of each gene set are shown in Supplementary Tables S1, S4.

We applied BISG on the datasets of brain lower grade glioma, lung adenocarcinoma and breast invasive carcinoma from the cBioPortal database (Table 1). Under the default and the same parameter setting as in Su et al. (2019), we identified 24, 7, and 6 significant cancer survival-related biomarker gene sets for lower grade glioma, lung adenocarcinoma and breast invasive carcinoma, respectively (as shown in Figure 3, and Supplementary Figures S1, S2 and Supplementary Table S4). The identified gene sets include 109, 82, and 58 genes, respectively. Multiple cancer survival-related genes were found in these genes, including CDH17 (Qiu et al., 2019), PTPRJ (D'Agostino et al., 2018), SLC16A14 (Elsnerova et al., 2017), TMTC2 (He et al., 2018), and NOTCH4 (Wang et al., 2018). Moreover, the results of gene set enrichment analysis and pathway analysis showed that most of the genes have known involvement in cancers. The survival curves of patients with (over 80% bicluster genes significantly upregulated) and without (others) top-ranked four most significant biclusters for each of the three cancer types are shown in Supplementary Figure S3, where the bicluster gene sets identified by our methods can well separate patients into two different survival groups.

### System Analysis Survival-Related Biomarker Gene Sets in 12 Different Cancer Types

**Figure 4:**
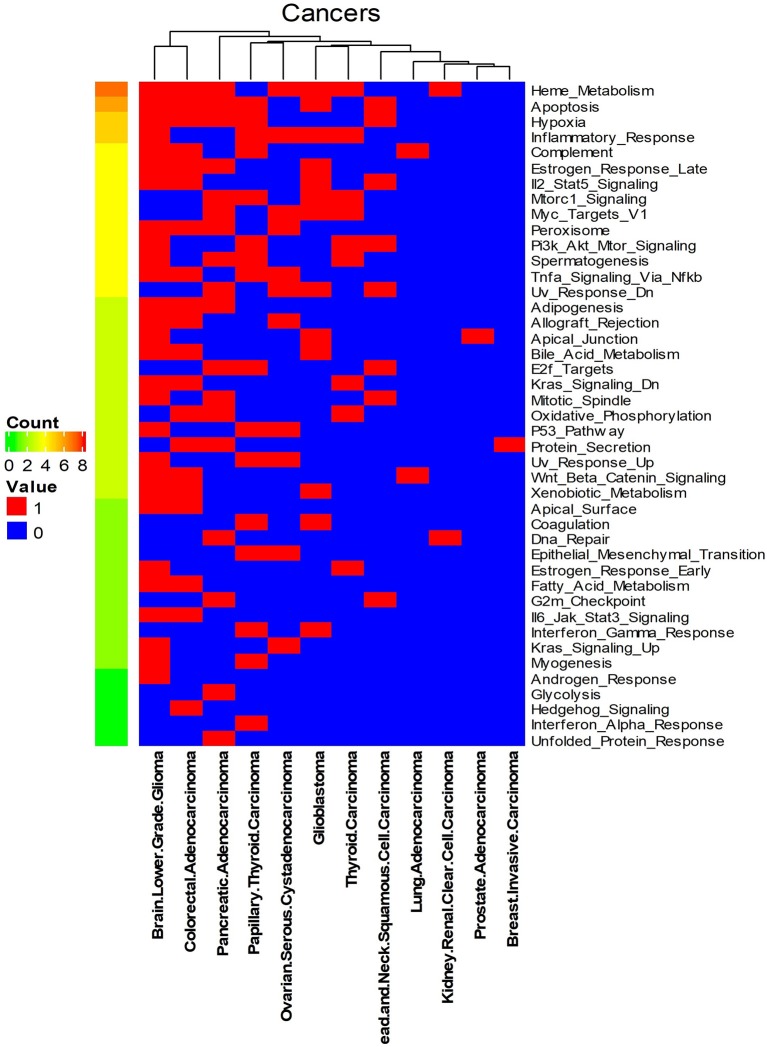
Enriched GSEA hallmark gene sets of all the biomarker gene sets of all the 12 cancer types. Names on the right Y-axis are the hallmark gene sets. Names on the bottom X-axis are the names of the 12 cancer types. Count means the number of cancers whose significant gene sets enriched in corresponding hallmark gene sets. Values in this figure are 0 or 1. Zero means the biomarker gene sets of the corresponding cancer are not enriched in the hallmark gene sets.

We systematically detected significant survival-related biomarker genes sets in 12 different cancer types with datasets in Table 1. The number of significant biomarker gene sets and their corresponding gene IDs for each cancer are shown in Supplementary Table S2. To find their relationships and functions of these significant biomarker gene sets, firstly, we conducted a function enrichment analysis with the GSEA hallmark gene sets from MSigDB (Liberzon et al., 2015). As shown in Figure 4, the function enrichment is mostly in heme metabolism, apoptosis, hypoxia, and inflammatory response. These are consistent with current findings. For example, according to Kalainayakan et al. (2019), cyclopamine tartrate suppresses tumor growth in the lung by inhibiting heme metabolism and OXPHOS (oxidative phosphorylation). A hallmark of cancer is the ability of malignant cells to evade apoptosis (Hanahan and Weinberg, 2011). Avoiding apoptosis is integral to tumor development and resistance to therapy. According to Muz et al. (2015), hypoxia stimulates a complex cell signaling network in cancer cells, including the HIF, PI3K, MAPK, and NFγB pathways. According to Nishijima et al. (2019), inflammatory markers are predictive of poorer survival, independent of traditional prognostic factors in older adults with cancer.

We also analyzed the enriched KEGG pathways of all the bicluster gene sets. As shown in Supplementary Figure S4, focal adhesion, neuroactive ligand receptor interaction, endocytosis and pathways in cancer are the most commonly enriched pathways by these gene sets. Finally, we systematically analyzed gene family information of all the biomarker gene sets of each cancer type. Results were shown in Supplementary Table S3. According to our analysis, genes in all the survival-related gene sets mainly from five gene families: microRNA protein-coding host genes, zinc fingers C2H2-type, solute carriers, CD molecules and ankyrin repeat domain-containing genes. Many of these genes are known survival-related (detailed information and the corresponding literature are shown in Supplemental Material). Furthermore, we found that many cancer survival-related genes identified so far are also from these gene families. For example, LEMD1 and EPHB2 are microRNA protein coding host genes, and SLC2A3 from solute carriers (Martinez-Romero et al., 2018). Other two survival-related genes RAD21 and CKS2 are microRNA protein coding host genes (van't Veer et al., 2002). In addition, CDH1 is from CD molecule (Gao et al., 2019). Of the 68 cancer survival-related gene sets in Varn et al. (2015), HMMR from CD molecules, MCM7 and CKS2 are microRNA protein coding host genes. Of the 129 ovarian cancer survival-related genes in Shen et al. (2019), 17 are from CD molecules gene family, 7 from microRNA protein-coding host genes, 1 from ankyrin repeat domain-containing gene family.

### Results Independent Tests

**Figure 5:**
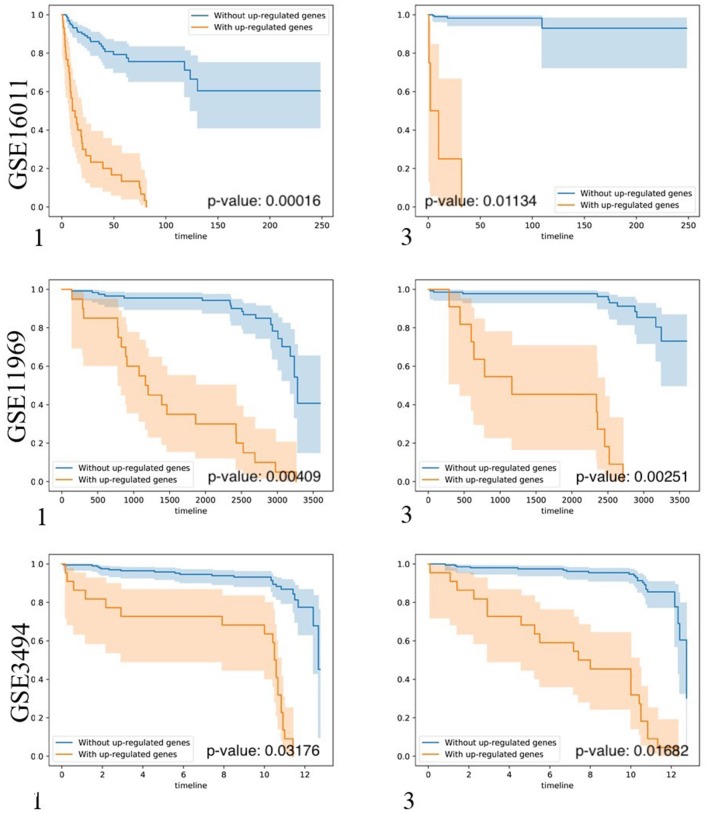
Kaplan-Meier plots of the survival analysis of the samples from brain lower grade glioma (GSE16011), lung adenocarcinoma (GSE11969), and breast invasive carcinoma (GSE3494) patients. 1, 3 means the first and the third top-ranked biomarker gene sets detected by BISG with corresponding cBioPortal datasets. The patients were separated into two groups according to the expression profiles of biomarker genes in the selected biomarker gene set. These genes provided the best split between patients of high and low risk based on their expression levels. In the case of genes in biomarker gene sets (labeled in brown) the over-expression is correlated with poor survival (only up-regulated genes were considered); and in the case of patients without biomarker genes (labeled in blue) the over-expression is correlated with good survival. In all cases the adjusted *p*-values of the analyses are highly significant, indicating that the two populations represented by the two curves have a very clear difference in their overall survival.

To test whether biomarker gene sets detected by BISG with datasets from cBioPortal database can differentiate patients into different survival groups with new independent datasets, we collected three microarray datasets GSE16011, GSE3494, and GSE11969, as well as their corresponding sample survival information (Table 2) from GEO as independent test datasets to confirm the biomarkers detected in gliomas, breast cancer and lung adenocarcinoma, respectively. For comparison, we selected the top-ranked first and third biomarker gene sets (as shown in Figure 3, and Supplementary Figures S2, S3) for each of the three cancer types. For any selected biomarker gene set, patients can be separated into two groups, one group with biomarker genes significantly changed, and the other with bicluster genes express normally. For survival analysis, we randomly selected the same number of patients from the two groups and test whether their survival curves are significantly different. As shown in Figure 5, the biomarker genes can well separate patients into different survival groups.

### Comparison With GSAS and IPSOV

**Figure 6:**
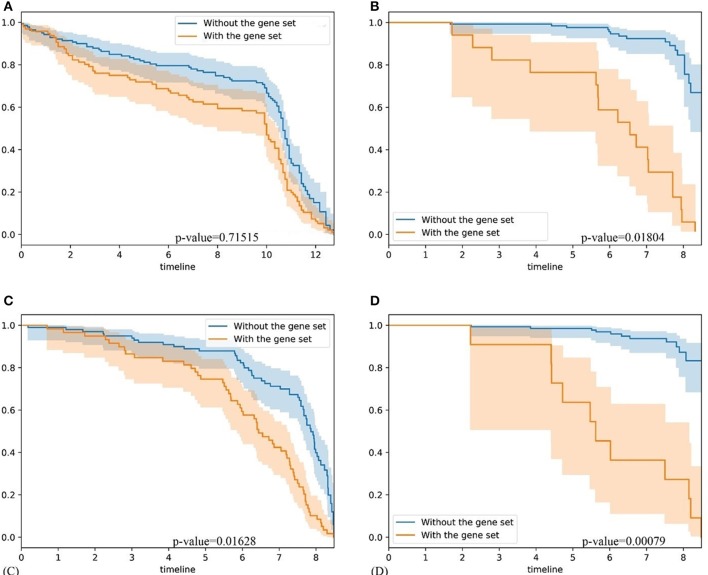
Comparison of gene set based patient survival group classification. “With gene set” means patients with over 80% expression of genes in the gene set significantly changed. “Without the gene set” means patients with the expression of genes in gene set are normal. **(A)** The survival curve of core gene set identified by the GSAS algorithm applied on the GSE1456 dataset. **(B)** The survival curve of the top-ranked gene set identified by our method applied on the GSE1456 dataset. **(C)** The survival curve of core gene set identified by the GSAS algorithm applied on the GSE3494 dataset. **(D)** The survival curve of the top-ranked gene set identified by our method applied on the GSE3494 dataset.

To further validate our method, firstly, we compared our methods with GSAS. GSAS quantitatively assesses a gene set's activity score with the BASE algorithm (Cheng et al., 2007), along with patient time-to-event data, to perform survival analyses to identify the gene sets that are significantly correlated with patient survival. Different from our method, they got gene sets directly from MSigDB. By applying on seven independent datasets, one core gene set with 68 genes were filtered out as most related to breast cancer survival. For comparison, we test whether the core gene set detected by GSAS and the top-ranked gene set identified by BISG with breast cancer datasets from cBioPortal database can different samples in GSE1456 (used by GSAS but not BISG) and GSE3494 (new to both two methods) into different survival groups. We run each method many times, and each time we randomly selected the same number of genes from their respective gene sets. The best performing results of each method are shown in Figure 6, where the gene set identified by BISG can better separate patients into different survival groups. In Figures 6A,C, patients with and without the biomarker genes based on GSAS have similar survival rates, while as shown in (B) and (D), the patients with biomarker genes identified by BISG have different survival rates from the rest. In this comparison, all the datasets are new and independent data that were not used in training BISG. Results indicate that the gene sets identified by BISG can better separate patients into different survival groups.

Furthermore, we also compared BISG with IPSOV. We tested whether the ovarian cancer survival-related gene sets detected by IPSOV (with data from GSE32062) and the top-ranked gene set identified by BISG with ovarian cancer datasets from the cBioPortal database can differentiate samples in GSE32062 (used by GSAS but not BISG) into different survival groups. Detailed results are shown in Supplementary Figure S5. Results showed that the biomarker gene set identified by BISG can better separate patients into different survival groups. Again, all the samples for comparison with GSAS were not used by BISG for the selection of biomarker gene sets, which means the biomarker genes identified by BISG are more likely cancer survival related genes.

Based on the fast GPU implementation of the RFN model, BISG can do biclustering analysis of large input datasets in a fast and accurate way, which enables BISG using a multi-sampling strategy to iteratively detect survival-related biomarker gene sets. In contrast to the standard clustering, the samples of a bicluster are only similar to each other on a subset of genes. As a result, genes in each significant bicluster can better differentiate samples into different survival groups. Compared with GSAS and IPSOV, the biomarker gene sets of our method are directly detected from biclustering analysis of the expression datasets, which can well capture the dynamic change of gene sets, and can reflect the real relationships of these genes.

## Conclusion

In this paper, we proposed BISG for identifying cancer survival-related biomarker gene sets. BISG can efficiently conduct biclustering for high-dimensional gene expression matrix, and along with patient time-to-event data perform survival analyses. To speed up computation, BISG performs a generalized alternating minimization algorithm with GPU implementations. In this way, BISG can efficiently construct very sparse, non-linear, high-dimensional representations of the input via their posterior means. To identify robust biomarker gene sets, multiple iterations and a random sampling strategy were utilized, and each time only bicluster genes that can significantly differentiate patient survival groups were kept. To detect patterns in survival-related gene sets, we systematically analyzed 12 different cancer types, and identified their enriched pathways and their gene families. The results indicated that the identified gene families and genes are biologically meaningful and consistent with the existing scientific findings. With several independent test datasets, identified biomarkers were confirmed. We also compared BISG with two related methods, and BISG outperformed them. The predicted biomarker gene sets can be further investigated for improving cancer patient survival. BISG is now based on a simple factor analysis model, which can be further extended into multi-layers with a deep learning network structure.

Our method has the potential to be extended for single-cell RNA-seq analysis, which has been widely applied in studying cell heterogeneity such as cells of different cancer types or subtypes. A pertinent question in such analyses is to identify cell subpopulations. Our methods can conduct biclustering effectively and efficiently especially for big expression matrices. Ongoing consortium efforts have generated extensive atlases of single-cell datasets covering diverse biological contexts with thousands of samples (Xie et al., 2019), and our methods may be suitable for analyzing them. We will explore applications of our method on single-cell RNA-seq analyses as our future work.

## Data Availability Statement

Publicly available datasets were analyzed in this study. This data can be found here: GSE3439, GSE11969, GSE16011, GSE1456, and GSE32062, https://www.cbioportal.org/.

## Author Contributions

LS, DX, and GL contributed conception and design of the study. LS, JW, and JG downloaded and organized datasets. LS performed the statistical and result analysis. LS wrote the first draft of the manuscript. All authors contributed to manuscript revision, read, and approved the submitted version.

## Conflict of Interest

The authors declare that the research was conducted in the absence of any commercial or financial relationships that could be construed as a potential conflict of interest.

## References

[B1] Blei D. M. Kucukelbir A. McAuliffe J. D. (2017). Variational inference: a review for statisticians. J. Am. Stat. Assoc. 112, 859–877. 10.1080/01621459.2017.1285773

[B2] Cerami E. Gao J. Dogrusoz U. Gross B. E. Sumer S. O. Aksoy B. A. (2012). The cBio cancer genomics portal: an open platform for exploring multidimensional cancer genomics data. Cancer Discov. 2, 960–960. 10.1158/2159-8290.Cd-12-0326 22588877 PMC3956037

[B3] Cheng C. Yan X. Sun F. Li L. M. (2007). Inferring activity changes of transcription factors by binding association with sorted expression profiles. BMC Bioinformatics 8:452. 10.1186/1471-2105-8-452 18021409 PMC2194743

[B4] Clevert D. A. Mayr A. Unterthiner T. Hochreiter S. (2015). Rectified factor networks. Adv. Neural. Inf. Process. Syst. 28:2028 10.5555/2969442.2969447

[B5] D'Agostino S. Lanzillotta D. Varano M. Botta C. Baldrini A. Bilotta A.. (2018). The receptor protein tyrosine phosphatase PTPRJ negatively modulates the CD98hc oncoprotein in lung cancer cells. Oncotarget 9, 23334–23348. 10.18632/oncotarget.25101 29805737 PMC5955124

[B6] Elsnerova K. Bartakova A. Tihlarik J. Bouda J. Rob L. Skapa P.. (2017). Gene expression profiling reveals novel candidate markers of ovarian carcinoma intraperitoneal metastasis. J. Cancer 8, 3598–3606. 10.7150/jca.20766 29151946 PMC5687176

[B7] Ganchev K. Graca J. Gillenwater J. Taskar B. (2010). Posterior regularization for structured latent variable models. J. Mach. Learn. Res. 11, 2001–2049. 10.5555/1756006.1859918

[B8] Gao C. D. Zhuang J. Zhou C. Li H. Y. Liu C. Liu L. J.. (2019). SNP mutation-related genes in breast cancer for monitoring and prognosis of patients: a study based on the TCGA database. Cancer Med. 8, 2303–2312. 10.1002/cam4.2065 30883028 PMC6537087

[B9] Gao J. J. Aksoy B. A. Dogrusoz U. Dresdner G. Gross B. Sumer S. O.. (2013). Integrative analysis of complex cancer genomics and clinical profiles using the cBioPortal. Sci. Signal. 6:pl1. 10.1126/scisignal.2004088 23550210 PMC4160307

[B10] Goel M. K. Khanna P. Kishore J. (2010). Understanding survival analysis: kaplan-meier estimate. Int. J. Ayurveda Res. 1, 274–278. 10.4103/0974-7788.76794 21455458 PMC3059453

[B11] Gravendeel L. A. M. Kouwenhoven M. C. M. Gevaert O. de Rooi J. J. Stubbs A. P. Duijm J. E.. (2009). Intrinsic gene expression profiles of gliomas are a better predictor of survival than histology. Cancer Research. 69, 9065–9072. 10.1158/0008-5472.Can-09-2307 19920198

[B12] Gunawardana A. Byrne W. (2005). Convergence theorems for generalized alternating minimization procedures. J. Mach. Learn. Res. 6, 2049–2073. 10.5555/1046920.1194913

[B13] Hanahan D. Weinberg R. A. (2011). Hallmarks of cancer: the next generation. Cell 144, 646–674. 10.1016/j.cell.2011.02.013 21376230

[B14] He R. Q. Zhou X. G. Yi Q. Y. Deng C. W. Gao J. M. Chen G.. (2018). Prognostic signature of alternative splicing events in bladder urothelial carcinoma based on spliceseq data from 317 cases. Cell Physiol. Biochem. 48, 1355–1368. 10.1159/000492094 30048970

[B15] Hochreiter S. Bodenhofer U. Heusel M. Mayr A. Mitterecker A. Kasim A.. (2010). FABIA: factor analysis for bicluster acquisition. Bioinformatics 26, 1520–1527. 10.1093/bioinformatics/btq227 20418340 PMC2881408

[B16] Kalainayakan S. P. Ghosh P. Dey S. Fitzgerald K. E. Sohoni S. Konduri P. C.. (2019). Cyclopamine tartrate, a modulator of hedgehog signaling and mitochondrial respiration, effectively arrests lung tumor growth and progression. Sci. Rep. 9:1405. 10.1038/s41598-018-38345-1 30723259 PMC6363760

[B17] Koyuturk M. Szpankowski W. Grama A. (2004). Biclustering gene-feature matrices for statistically significant dense patterns, in 2004 IEEE Computational Systems Bioinformatics Conference Proceedings (Stanford, CA), 480–484.

[B18] Liberzon A. Birger C. Thorvaldsdottir H. Ghandi M. Mesirov J. P. Tamayo P. (2015). The Molecular Signatures Database (MSigDB) hallmark gene set collection. Cell Syst. 1, 417–425. 10.1016/j.cels.2015.12.004 26771021 PMC4707969

[B19] Martinez-Romero J. Bueno-Fortes S. Martin-Merino M. de Molina A. R. de Las Rivas J. (2018). Survival marker genes of colorectal cancer derived from consistent transcriptomic profiling. BMC Genomics 19:857. 10.1186/s12864-018-5193-9 30537927 PMC6288855

[B20] Muz B. de la Puente P. Azab F. Azab A. K. (2015). The role of hypoxia in cancer progression, angiogenesis, metastasis, and resistance to therapy. Hypoxia 3, 83–92. 10.2147/HP.S93413 27774485 PMC5045092

[B21] Nishijima T. F. Deal A. M. Lund J. L. Nyrop K. A. Muss H. B. Sanoff H. K. (2019). Inflammatory markers and overall survival in older adults with cancer. J. Geriatr. Oncol. 10, 279–284. 10.1016/j.jgo.2018.08.004 30131235

[B22] Padilha V. A. Campello R. J. G. B. (2017). A systematic comparative evaluation of biclustering techniques. BMC Bioinformatics 18:55. ARTN 55 10.1186/s12859-017-1487-1 28114903 PMC5259837

[B23] Palazon A. Tyrakis P. A. Macias D. Velica P. Rundqvist H. Fitzpatrick S.. (2017). An HIF-1alpha/VEGF-A axis in cytotoxic T cells regulates tumor progression. Cancer Cell 32, 669–683. 10.1016/j.ccell.2017.10.003 29136509 PMC5691891

[B24] Pawitan Y. Bjohle J. Amler L. Borg A. L. Egyhazi S. Hall P.. (2005). Gene expression profiling spares early breast cancer patients from adjuvant therapy: derived and validated in two population-based cohorts. Breast Cancer Res. 7, R953–R964. 10.1186/bcr1325 16280042 PMC1410752

[B25] Qiu H. B. Zhang L. Y. Ren C. Zeng Z. L. Wu W. J. Luo H. Y.. (2019). Targeting CDH17 suppresses tumor progression in gastric cancer by downregulating Wnt/beta-catenin signaling. PloS ONE 14:e56959. 10.1371/journal.pone.0056959 23554857 PMC3598811

[B26] Saelens W. Cannoodt R. Saeys Y. (2018). A comprehensive evaluation of module detection methods for gene expression data. Nat. Commun. 9:1090. 10.1038/s41467-018-03424-4 29545622 PMC5854612

[B27] Shen S. P. Wang G. R. Zhang R. Y. Zhao Y. Yu H. Yongyue W.. (2019). Development and validation of an immune gene-set based prognostic signature in ovarian cancer. EBioMedicine 40, 318–326. 10.1016/j.ebiom.2018.12.054 30594555 PMC6412087

[B28] Singh R. Mukhopadhyay K. (2011). Survival analysis in clinical trials: basics and must know areas. Perspect. Clin. Res. 2, 145–148. 10.4103/2229-3485.86872 22145125 PMC3227332

[B29] Srivastava N. Hinton G. Krizhevsky A. Sutskever I. Salakhutdinov R. (2014). Dropout: a simple way to prevent neural networks from overfitting. J. Mach. Learn. Res. 15, 1929–1958.

[B30] Su L. Liu G. Wang J. Xu D. (2019). A rectified factor network based biclustering method for detecting cancer-related coding genes and miRNAs, and their interactions. Methods 166, 22–30. 10.1016/j.ymeth.2019.05.010 31121299 PMC6708461

[B31] Takeuchi T. Tomida S. Yatabe Y. Kosaka T. Osada H. Yanagisawa K.. (2006). Expression profile-defined classification of lung adenocarcinoma shows close relationship with underlying major genetic changes and clinicopathologic behaviors. Int. J. Clin. Oncol. 24, 1679–1688. 10.1200/Jco.2005.03.8224 16549822

[B32] Theodosopoulos T. (2007). A reversion of the chernoff bound. Stat. Probabil. Lett. 77, 558–565. 10.1016/j.spl.2006.09.003

[B33] van't Veer L. J. Dai H. Y. van de Vijver M. J. He Y. D. D. Hart A. A. M. Mao M. (2002). Gene expression profiling predicts clinical outcome of breast cancer. Nature 415, 530–536. 10.1038/415530a 11823860

[B34] Varn F. S. Ung M. H. Lou S. K. Cheng C. (2015). Integrative analysis of survival-associated gene sets in breast cancer. BMC Med. Genomics 8:11. ARTN 11 10.1186/s12920-015-0086-0 25881247 PMC4359519

[B35] Wang J. W. Wei X. L. Dou X. W. Huang W. H. Du C. W. Zhang G. J. (2018). The association between Notch4 expression, and clinicopathological characteristics and clinical outcomes in patients with breast cancer. Oncol. Lett. 15, 8749–8755. 10.3892/ol.2018.8442 29805613 PMC5958688

[B36] Wang W. Liu W. (2018). Integration of gene interaction information into a reweighted random survival forest approach for accurate survival prediction and survival biomarker discovery. Sci. Rep. 8:13202. 10.1038/s41598-018-31497-0 30181543 PMC6123437

[B37] Xie J. Ma A. Zhang Y. Liu B. Cao S. Wang C.. (2019). QUBIC2: a novel and robust biclustering algorithm for analyses and interpretation of large-scale RNA-Seq data. Bioinformatics 36, 1143–1149. 10.1093/bioinformatics/btz692 31503285 PMC8215922

[B38] Xu L. Choy C. S. Li Y. W. (2016). Deep sparse rectifier neural networks for speech denoising, in 2016 IEEE International Workshop on Acoustic Signal Enhancement (Xi'an: Iwaenc).

[B39] Yoshihara K. Tsunoda T. Shigemizu D. Fujiwara H. Hatae M. Fujiwara H.. (2012). High-risk ovarian cancer based on 126-gene expression signature is uniquely characterized by downregulation of antigen presentation pathway. Clin. Cancer Res. 18, 1374–1385. 10.1158/1078-0432.Ccr-11-2725 22241791

[B40] Zhang Y. Xie J. Yang J. Y. Fennell A. Zhang C. Ma Q. (2017). QUBIC: a bioconductor package for qualitative biclustering analysis of gene co-expression data. Bioinformatics 33, 450–452. 10.1093/bioinformatics/btw635 28172469

