# Supplementary material for: Detecting Cancer Survival Related Gene Markers Based on Rectified Factor Network
Source: Front Bioeng Biotechnol. 2020 Apr 23;8:349. doi: 10.3389/fbioe.2020.00349 (PMC7212422; doi:10.3389/fbioe.2020.00349)
Supplement: Supplementary file 1 [file Data_Sheet_1.pdf]

Supplementary Materials

1 Supplementary Figures and Tables

1.1 Supplementary Figures

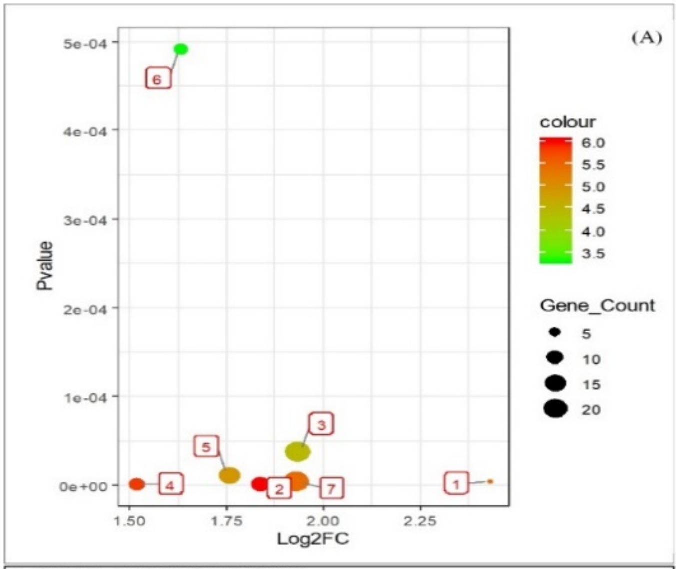

(B)

1: SFTPB,ADAMTSL3,DCTN1
2:
COL6A1,ABL2,TAP2,RASAL2,HNRNPD,DIAPH3,OAS2,LMNB2,S
HCBP1,TACC3,SPTAN1
3:
NME7,EVC2,FLNC,COL6A1,VEGFC,LUZP1,MMP14,SRPX2,ABL2,
CD109,IGF2BP2,IDS,NRP2,NCAPG2,RASAL2,LYAR,MKI67,ARHG
AP10,MDFIC,AIM1,MKLN1,BRAF,CAMSAP1,TNPO3
4:
RALGAPB,ZNF347,DAAM1,CEP350,PIK3CA,ZC3HAV1,HNRNPK,
TRIP11
5:
SCNN1A,GPR39,AAMP,P4HB,MYCT1,VPS52,NOTCH4,ACE,MMR
N1,DCLK3,FLT1,TMEM63B,PRSS16,DCTN1,AHCYL2,ARHGEF10
6: EPAS1,GPR39,MYCT1,ADAMTSL3,NOTCH4,SEMA3G,MMRN1
7:
ATPIF1,SLC25A38,EDEM2,PPP1R16A,SCNN1A,MFSD3,DUSP22,T
UFM,GPR39,ADI1,RILP,SLC41A3,CYP4B1,SMPDL3B,LPAR1,FHI
T,SELENBP1,PNPLA4,TSTA3,HADHB,ALDH3B1,TBC1D2,SERPI
NI1,WDR89

Supplementary Figure S1 | Significant survival-related gene sets detected in lung adenocarcinoma. (A) Gene set fold change and p-value distribution. (B) Gene names of the 7 gene sets.

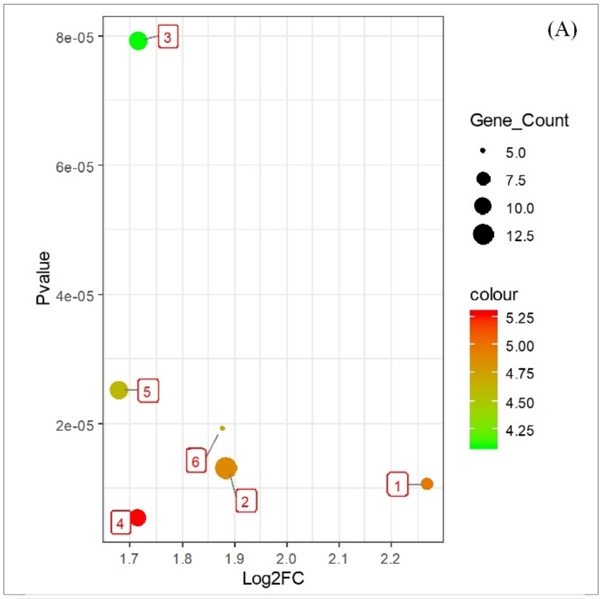

(B)

1: CYTH3,MBOAT4,ARRDC1,MROH8,MTOR,INADL,PLD2
2:
C2CD3,DMXL1,TAB2,ZSWIM5,CHRNA9,ANKRD50,AGTR2,LARP7,
ATP8B1,SUPT5H,HDAC8,KDM4A,FBXO44,CMTR2
3:
ETS2,MTG2,WISP1,TMTC2,REPIN1,TRIM33,TRADD,PGM1,AGBL5,J
DP2,UQCR10
4:
CHRNA2,PDZRN3,CCDC181,SLC12A1,SORL1,CACNG3,FAM214A,M
ELK,HIST1H2BL,RNF219
5:
MYO5A,ZFP28,FOXN3,ETNK1,LPAR4,SPATA31D5P,NAPA,DUSP27,
PRKAA1,DGKH,HIST1H3D
6: SDK2,WDR72,UGT1A4,USP6NL,EXOSC10

Supplementary Figure S2 | 6 Significant survival-related gene sets detected in breast invasive carcinoma. (A) Gene set fold change and p-value distribution. (B) Gene names of the 6 gene sets.

We compared the performance of the gene set identified by IPSOV in (Shen et al. 2019; Yoshihara et al. 2012) and the top-ranked gene set identified by our method with the same dataset (GSE32062) used in (Shen et al. 2019; Yoshihara et al. 2012). IPSOV used the immune genes from the ImmPort database to develop an immune-based prognostic score for OV (Ovarian cancer) (Shen et al. 2019). By systemically analyzing 17 public microarray datasets, 129 genes from 15 immune categories were associated with overall survival among the 1905 immune-related microarray probes. Of the 129 genes, 3 genes (ID4, MTRF1 and SLC7A11) were also confirmed associated with survival in (Yoshihara et al. 2012). For comparison, we randomly selected the same number of genes from the 129 genes (each time including ID4, MTRF1 and SLC7A11) as the top-ranked survival related gene set identified by BISG with TCGA ovarian cancer dataset.

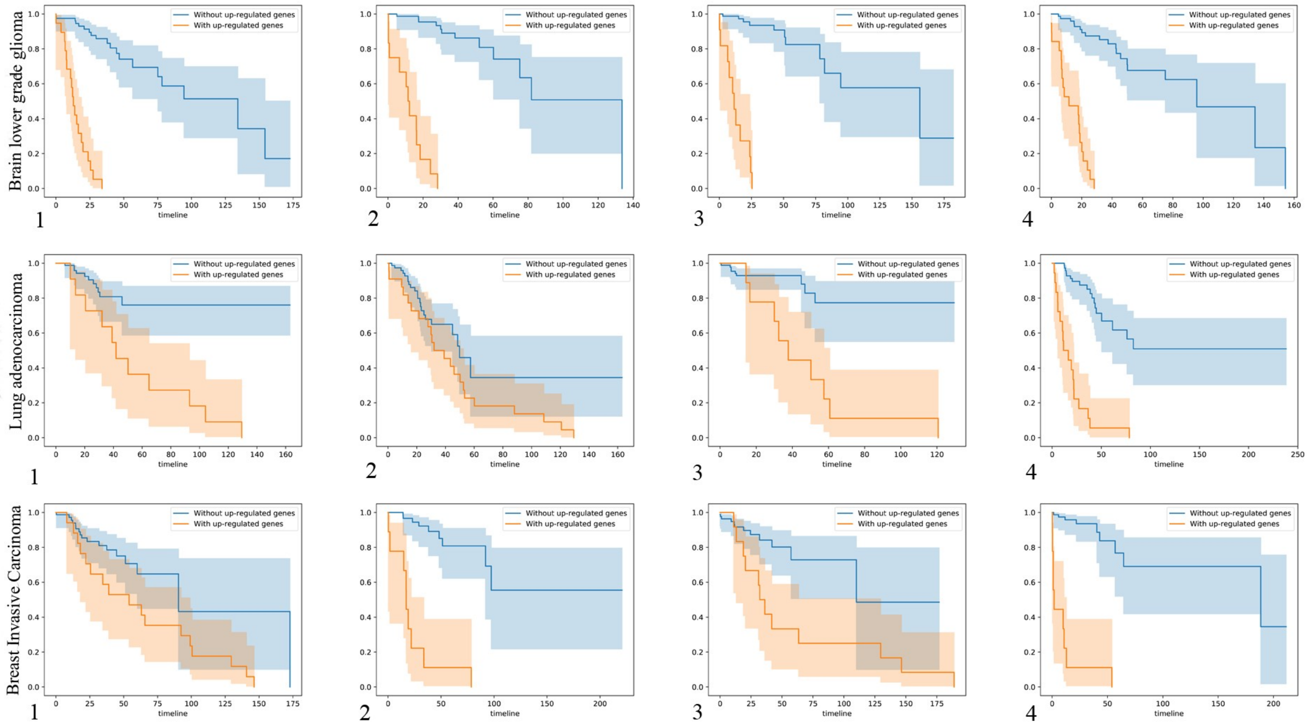

Supplementary Figure S3 | Survival curves of patient groups with and without changed expression of the biomarker gene sets. In this figure, 1,2,3,4 means the top-ranked four biomarker genes sets. The top four figures are the survival curves of brain lower grade glioma patients with and without the top-ranked bicluster genes. The middle and bottom level figures are survival curves of lung adenocarcinoma and breast invasive carcinoma, respectively.

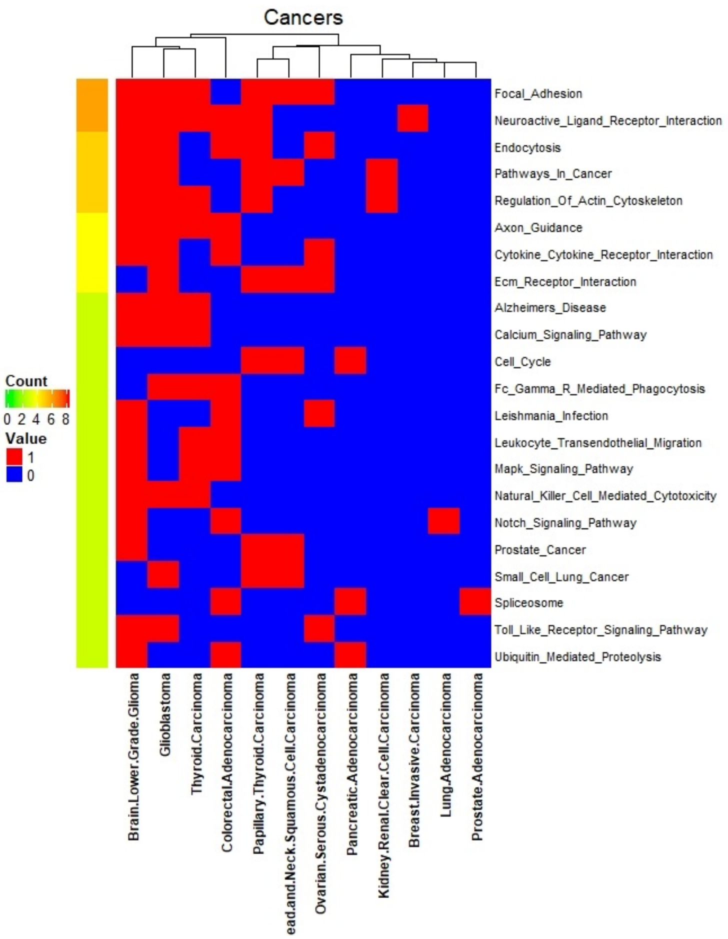

Supplementary Figure S4 | Enriched KEGG pathways of significant gene sets of all the 12 cancer types. Names on the right Y-axis are the official KEGG pathway names. Names on the bottom X-axis are the names of the 12 cancer types. Count means the number of cancers whose significant gene sets enriched in the corresponding KEGG pathway. Values in this figure are 0 or 1. 0 means the gene sets of the corresponding cancer are not enriched in the KEGG pathway.

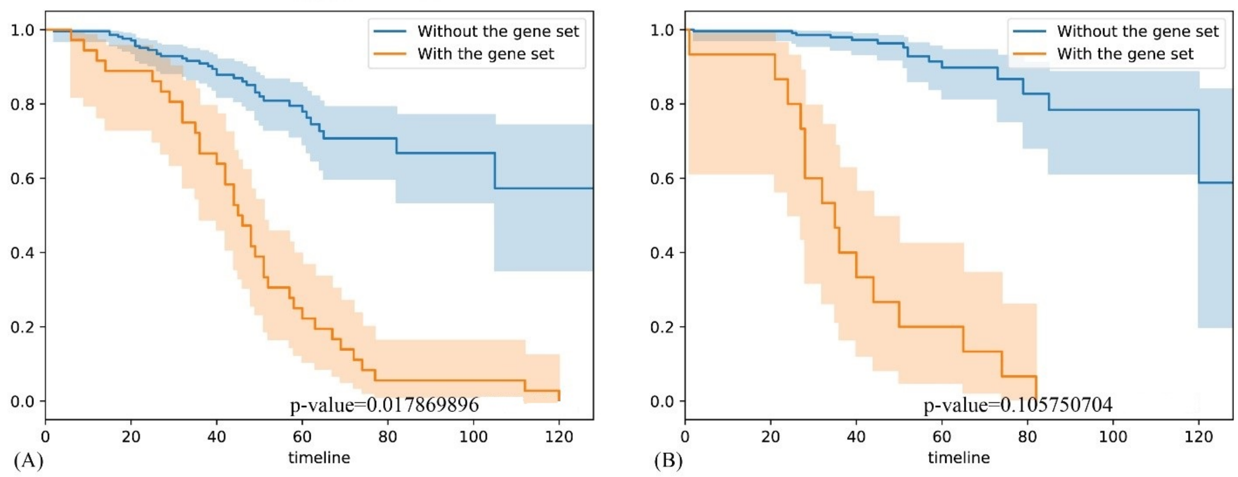

Supplementary Figure S5 | Comparison of gene set based patient survival group classification. (A) Survival curve of the gene set identified by IPSOV algorithm applied on the GSE32062 dataset. (B) Survival curve of the top-ranked gene set identified by our method applied on the GSE32062 dataset.

1.2 Supplementary Tables

Supplementary Table S1 | The corresponding gene names of each gene sets in Figure 3.

ID	Gene names
1	RPE65, ARHGEF16, PLBD2, ZNF711, TNNT1, PTPRJ, PPFIA3
2	USP51, PCSK6, ARHGEF16, PLXNA2
3	TRPM6, FZD10, ROS1, GK, PTPRJ, NEK8
4	SLC16A14, TNFRSF10C, PCSK6, OR5H2, SLC9C2, GALNTL6, PDGFRA, AURKC
5	TRIP12, EIF2B4, PCSK6, PDGFRA, NTN3, CCDC84
6	PCSK6, PTPRJ, PPFIA3
7	ARHGEF16, EMILIN2, FDCSP, GLB1L, RAG2, JAM3, SYCP2L, DNMT3B, PLXNA2
8	SLC16A14, TNFRSF10C, ZBTB20, TSHR, F5, DNMT3B, PTPRJ, AURKC, PLXNA2, SGSM2
9	LETM1, ARHGEF16, ZNF688, ZNF711, PLXNA2

10	RPE65, PCSK6, GK, PLCB2, PDGFRA, PTPRJ, PLXNA2
11	TRIP12, TSPAN33, SLC16A4, PDGFRA
12	B4GALT1, FASLG, KCNJ15, RLIM, SPINT1, NLRP14, ZBTB20, ROS1, KIAA1161, CENPK, PTPRJ, AP4B1, PLXNA2
13	DHTKD1, DYX1C1, SSBP2, DCAF12L1, ANO7
14	TNFRSF10C, LETM1, SLC26A2, PCSK6, JMJD1C, ITGA10, NTN3, PTPRJ
15	ASTN1, KIAA1147, RRNAD1, DYSF, ITPR3, IQCH, EMILIN2, PLBD2, DMRTC2, NLRP2, WBSCR17, PLXNA2
16	TNFRSF10C, PCSK6, F5, CENPK, GALNTL6, AURKC, PLXNA2
17	ZNF592, DHTKD1, DMC1, RIOK3, TTC14
18	TNFRSF10C, TRIP12, SLC26A2, PCSK6, FKBP9, CDH20, GPR55, ZNF688, HMGA2, ALPP, SFMBT2, GK, GALNTL6, PLCB2, TNNT1, PDGFRA, NTN3, PTPRJ, PLXNA2
19	TNFRSF10C, LETM1, CDH20, GPR55, ZNF688, SYNE3, ITGA10, F5, CENPK, TNNT1, DSG1, NTN3, PTPRJ, MPO, AURKC, PLXNA2, ZSCAN5A
20	LDB1, UGGT1, RBP3, LETM1, UTRN, JAG2, MKRN1, SIGLEC6, NOTCH4, EMILIN2, CDH17, CLIC4, FDCSP, CLDN14, MAP3K19, ALPP, TESK2
21	TRRAP, DDX31, TTC17, TP53BP1, EMILIN2, MEGF8, OR5H2, CNTN2, TNNT3, RIMBP2, AURKC, PLXNA2
22	TNFRSF11A, TP53BP1, GPR17, TYR, SEMA6C, PLXNA2
23	GAPVD1, PCSK6, FDCSP, NTN3
24	CNTRL, TNNT1, PTPRJ, AP4B1

Supplementary Table S2 | The number of significant survival related gene sets and their corresponding number of genes included.

ID	Cancer name	Gene sets number	Gene number
**1**	Brain Lower Grade Glioma	24	109
**2**	Colorectal Adenocarcinoma	41	626
**3**	Glioblastoma	15	122
**4**	Head and Neck Squamous Cell Carcinoma	8	108
**5**	Kidney Renal Clear Cell Carcinoma	12	143
**6**	Lung Adenocarcinoma	7	82
**7**	Ovarian Serous Cystadenocarcinoma	8	54
**8**	Pancreatic Adenocarcinoma	8	81
**9**	Papillary Thyroid Carcinoma	19	44
**10**	Prostate Adenocarcinoma	3	27
**11**	Thyroid Carcinoma	23	56
**12**	Breast Invasive Carcinoma	6	58

Supplementary Table S3 | Top 5 gene families mostly enriched by genes in survival-related gene sets. “NA” means no genes located in the corresponding gene family.

ID	Cancer name	MicroRNA protein coding host genes	Zinc fingers C2H2-type	Solute carriers	CD molecules	Ankyrin repeat domain containing
**1**	Brain Lower Grade Glioma	PTPRJ, CDH13, ZBTB20, COL18A1, CAPN15, ST3GAL3	ZNF688, ZBTB20, ZNF711	LETM1, SLC16A14, SLC16A4, SLC25A36, SLC30A6, SLC26A2,	PTPRJ, TNFRSF10C, PDGFRA, TNFRSF11A, FASLG	EHMT1, NOTCH4

				SLC9C2		
**2**	Colorectal Adenocarcinoma	RTN1, ITCH, SEMA3F, SLC35B2, LDLRAD3, FOCAD	ZNF671, ZNF57, ZKSCAN2, ZNF234	SLC35B2, SLC6A2, SLC9A7	LEPR, FCGR2B, RHCE, CDH5	CTTNBP2, TNKS2, NFKBIE, ANKRD55
**3**	Glioblastoma	PLD3	ZNF674	MAGT1, SLC22A18, SLC9A7, SLC9A4	ITGA3, TNFSF10	ASAP2
**4**	Head and Neck Squamous Cell Carcinoma	CDH23	ZNF641, ZNF473, ZNF324B, ZNF619, IKZF4	NA	NA	TRPV5
**5**	Kidney Renal Clear Cell Carcinoma	NA	ZNF692, MAZ	NA	NA	ANKS3, DAPK1
**6**	Lung Adenocarcinoma	NA	NA			NOTCH4
**7**	Ovarian Serous Cystadenocarcinoma	TNS1, SLC12A8	ZEB2	SLC12A8	TLR4	NA
**8**	Pancreatic Adenocarcinoma	HNRNPK	ZNF544, ZNF707, GLIS3	NA	NA	NA

**9**	Papillary Thyroid Carcinoma	GRID1,<br>MAST1	ZNF580,<br>ZNF543	SPNS1,<br>SLC5A2,<br>SLC40A1	FCRL2	ANKS4B,<br>ANKRD13D
**10**	Prostate Adenocarcinoma	NA	NA	NA	NA	NA
**11**	Thyroid Carcinoma	TNS1,<br>VAV3	ZBTB24	NA	NA	NA
**12**	Breast Invasive Carcinoma	DMXL1	NA	NA	NA	NA

The detailed analysis of each gene family and their corresponding genes are as follows:

MicroRNA protein-coding host genes: These miRNA host genes encode proteins with a broad spectrum of biological roles ranging from embryonic development, to the cell cycle, and physiology. According to the published results, many survival-related genes belong to these gene families. For example, the non-small cell lung cancers (NSCLCs) of patients showing a short survival rate express the lowest and the highest levels of PTPRJ and SLC3A (D'Agostino et al. 2018). CDH13 down-regulation has been associated with poorer prognosis in various carcinomas, such as lung, ovarian, cervical and prostate cancer (Andreeva & Kutuzov 2010). Lastly, researchers found that ITCH expression is significantly upregulated in invasive and metastatic breast cancer cases and is associated with worse survival (Salah et al. 2014). Survival analyses using the OncoLnc database revealed that elevated TNS1 levels were associated with a poor overall survival in CRC patients (Zhou et al. 2018). Overall, VAV3 was overexpressed in human breast cancer cells and this correlated with a shorter survival time, indicating that VAV3 is a biomarker of a poor prognosis for breast cancer patients (Chen et al. 2015).

Zinc fingers C2H2-type: Zinc finger proteins are the largest transcription factor family in the human genome. Functions of zinc finger including development, differentiation, metabolism, autophagy and in cancer progression (Jen & Wang 2016). For example, the positive expression of ZBTB20 was associated with large tumor size, high Edmondson-Steiner grading and advanced tumor stage (Kan et al. 2016). Additionally, hepatocellular carcinoma patients with positive expression of ZBTB20 had a poorer 5-year survival. Survival analysis established that the downregulation of ZNF671 predicts poor prognosis in breast invasive carcinoma (BRCA), cervical squamous cell carcinoma and endocervical adenocarcinoma (CESC), head and neck squamous cell carcinoma (HNSC), kidney renal papillary cell carcinoma (KIRP), lung adenocarcinoma (LUAD), pancreatic adenocarcinoma (PAAD), and uterine corpus endometrial carcinoma (UCEC) solid tumors (Zhang et al. 2019). Collaboration between ZEB2 and Sp1 induces cancer cell survival and proliferation, endothelial cell activation, and tumor angiogenesis (Ko 2018). Other genes in this family including ZNF229, ZNF233, ZNF395 and ZNF432 are DNA-binding protein domains consisting of zinc fingers. Many of these zinc finger proteins, including ZNF233, have been found to be associated with the abnormality of chromosome 19 in the

studies of kidney and pancreatic cancers. Our analysis reveals that Zinc finger proteins and the corresponding pathway might be associated with the survival of pancreatic cancer.

Solute carriers (SLC family): The survival analysis indicated that most SLC gene members were significantly associated with overall survival (Ding et al. 2019). For instance, SLC22A8, SLC8A3, SLC24A6 are membrane transport proteins that are involved in the transport and excretion of many organic ions, drugs and toxicants. Some genes in SLC family are cancer-related, for example, SLC43A2 whose overexpression is associated with the adenocarcinomas and squamous cell carcinoma. LETM1 overexpression is correlated with the clinical features and survival outcome of breast cancer (Li et al. 2015). High expression of SLC16A14 gene is significantly associated with longer progression-free survival of patients (Elsnerova et al. 2017). Low expression of SLC22A18 predicts poor survival outcome in patients with breast cancer after surgery (He et al. 2011).

CD (cluster of differentiation) molecules: CD molecules are cell-surface molecules expressed on leukocytes and other cells relevant for the immune system. PDGFRA mutations and KIT exon 11 insertion or duplication mutations were associated with favorable RFS (recurrence-free survival), whereas KIT exon 9 mutations were associated with unfavorable outcome (Joensuu et al. 2017). High expression of ITGA3 promotes proliferation and cell cycle progression and indicates poor prognosis in intrahepatic cholangiocarcinoma (Huang et al. 2018). ITGAV, ITGA6, and ITGA3 as prognostic factors for disease-free survival of colorectal cancer (Linhares et al. 2015). Other results showed that FCRL2 expression was predictive of cancer overall survival (Shea et al. 2019). Accumulating evidence showed that high expression of TLR4 can act as a prognostic factor for survival in various cancers (Hao et al. 2018).

Ankyrin repeat domain-containing: This gene family plays diverse functions such as transcriptional initiators, cell-cycle regulators, cytoskeletal, ion transporters and signal transducers. The expression of EHMT1 was identified as an independent prognostic factor for overall survival in ESCC patients (Guan et al. 2014). NOTCH4 expression is associated with aggressive clinicopathological and biological phenotypes, and may predict poor prognosis in luminal breast cancer patients (Wang et al. 2018). Decreased expression of TRPV5/6 in tumor tissues was observed in NSCLC patients and was associated with shorter median survival time after surgical resection (Fan et al. 2014). Low levels of DAPK1 mRNA were associated with shorter survival in a liver cancer patient cohort, while negative staining of DAPK1 protein was significantly correlated with overall survival. Furthermore, DAPK1 was an independent prognostic marker for both progression and overall survival by multivariate analysis (Li et al. 2017).

Supplementary Table S4 | Detailed PubMed annotation of the genes in Supplementary Figures S1, S2 and Figure 3.

Gene Name	PubMed
AAMP	PMID: 23564791
ABL2	PMID: 24940071
ACE	PMID: 11041163
ADAMTSL3	PMID: 26462029
ADI1	PMID: 30858354
AGBL5	
AGTR2	

AHCYL2	PMID: 31799184
AIM1	PMID: 22402438
ALDH3B1	
ALPP	
ANKRD50	
ANO7	PMID: 30157291
AP4B1	
ARHGAP10	PMID: 20332263
ARHGEF10	
ARHGEF16	PMID: 21760942
ARRDC1	
ASTN1	
ATP8B1	
ATPIF1	
AURKC	PMID: 31142743
B4GALT1	PMID: 29793447
BRAF	PMID: 26396549
C2CD3	
CACNG3	PMID: 29242506
CAMSAP1	PMID: 26001296
CCDC181	PMID: 30498398
CCDC84	
CD109	PMID: 31316041
CDH17	PMID: 23554857
CDH20	PMID: 31998642
CENPK	PMID: 26587348
CEP350	
CHRNA2	
CHRNA9	
CLDN14	PMID: 27207647
CLIC4	PMID: 28205343
CMTR2	
CNTN2	
CNTRL	
COL6A1	PMID: 31268154
CYP4B1	
CYTH3	PMID: 27703978
DAAM1	PMID: 30911286
DCAF12L1	

DCLK3	
DCTN1	PMID: 29864111
DDX31	PMID: 29440146
DGKH	PMID: 30301218
DHTKD1	
DIAPH3	PMID: 26179371
DMC1	PMID: 25906155
DMRTC2	
DMXL1	
DNMT3B	PMID: 18414412
DSG1	
DUSP22	PMID: 26032091
DUSP27	
DYSF	
DYX1C1	PMID: 22375924
EDEM2	
EIF2B4	
EMILIN2	
EPAS1	PMID: 22848255
ETNK1	PMID: 31255331
ETS2	PMID: 28724426
EVC2	
EXOSC10	
F5	PMID: 29766637
FAM214A	
FASLG	
FBXO44	
FDCSP	
FHIT	
FKBP9	PMID: 31780055
FLNC	
FLT1	PMID: 25926745
FOXN3	PMID: 31214487
FZD10	
GALNTL6	
GAPVD1	
GK	
GLB1L	
GPR17	
GPR39	

GPR55	PMID: 27340777
HADHB	
HDAC8	PMID: 30519361
HIST1H2BL	PMID: 29184082
HIST1H3D	PMID: 28112369
HMGA2	PMID: 21252160
HNRNPD	PMID: 26318153
HNRNPK	PMID: 29262567
IDS	
IGF2BP2	PMID: 31852504
INADL	
IQCH	
ITGA10	PMID: 28540334
ITPR3	PMID: 26619122
JAG2	PMID: 31198409
JAM3	
JDP2	PMID: 28315425
JMJD1C	PMID: 29888107
KCNJ15	PMID: 30799948
KDM4A	PMID: 29113308
KIAA1147	
KIAA1161	
LARP7	PMID: 25053741
LDB1	PMID: 27713177
LETM1	PMID: 26722481
LMNB2	
LPAR1	PMID: 31384176
LPAR4	
LUZP1	
LYAR	PMID: 28686580
MAP3K19	
MBOAT4	
MDFIC	
MEGF8	PMID: 23063620
MELK	
MFSD3	
MKI67	PMC6775696
MKLN1	
MKRN1	

MMP14	PMID: 29552138
MMRN1	PMID: 25825478
MPO	PMID: 15705913
MROH8	
MTG2	
MTOR	
MYCT1	
MYO5A	
NAPA	
NCAPG2	PMID: 27862966
NEK8	PMID: 30333866
NLRP14	
NLRP2	
NME7	
NOTCH4	PMID: 29805613
NRP2	
NTN3	
OAS2	PMID: 30148861
OR5H2	
P4HB	PMID: 31467922
PCSK6	
PDGFRA	
PDZRN3	
PGM1	PMID: 30335765
PIK3CA	PMID: 22640628
PLBD2	
PLCB2	
PLD2	PMID: 24990948
PLXNA2	
PNPLA4	
PPFIA3	
PPP1R16A	
PRKAA1	PMID: 30253744
PRSS16	
PTPRJ	PMID: 29805737
RAG2	
RALGAPB	PMID: 22450745
RASAL2	PMID: 24029233
RBP3	
REPIN1	

RILP	
RIMBP2	
RIOK3	PMID: 25486436
RLIM	
RNF219	
ROS1	PMID: 29883837
RPE65	
RRNAD1	
SCNN1A	
SDK2	
SELENBP1	
SEMA3G	PMID: 18781179
SEMA6C	
SERPINI1	
SFMBT2	PMID: 27340776
SFTPB	
SGSM2	
SHCBP1	PMID: 27129942
SIGLEC6	
SLC12A1	
SLC16A14	PMID: 29151946
SLC16A4	
SLC25A38	
SLC26A2	PMID: 28108622
SLC41A3	
SLC9C2	
SMPDL3B	
SORL1	
SPATA31D5P	
SPINT1	
SPTAN1	PMID: 31186638
SRPX2	PMID: 24700475
SSBP2	PMID: 22472174
SUPT5H	
SYCP2L	PMID: 26362258
SYNE3	
TAB2	
TACC3	PMID: 26531241
TAP2	

TBC1D2	PMID: 32038997
TESK2	PMID: 29986997
TMEM63B	
TMTC2	PMID: 30048970
TNFRSF10C	PMID: 19035483
TNFRSF11A	
TNNT1	
TNNT3	
TNPO3	
TP53BP1	PMID: 28475402
TRADD	
TRIM33	
TRIP11	
TRIP12	PMID: 27425591
TRPM6	PMID: 30272358
TRRAP	PMID: 27066097
TSHR	PMID: 29344196
TSPAN33	
TSTA3	PMID: 26531722
TTC14	
TTC17	PMID: 30519852
TUFM	
TYR	PMID: 22045183
UGGT1	
UGT1A4	
UQCR10	
USP51	PMID: 29119051
USP6NL	PMID: 29691252
UTRN	
VEGFC	PMID: 18396396
VPS52	PMID: 28791438
WBSCR17	
WDR72	
WDR89	
WISP1	PMID: 30651114
ZBTB20	PMID: 26893361
ZC3HAV1	
ZFP28	
ZNF347	
ZNF592	

ZNF688	
ZNF711	
ZSCAN5A	
ZSWIM5	PMID: 30233241

References

Andreeva AV, and Kutuzov MA. 2010. Cadherin 13 in cancer. *Genes Chromosomes Cancer* 49:775-790. 10.1002/gcc.20787

Chen X, Chen S, Liu XA, Zhou WB, Ma RR, and Chen L. 2015. Vav3 oncogene is upregulated and a poor prognostic factor in breast cancer patients. *Oncology Letters* 9:2143-2148. 10.3892/ol.2015.3004

D'Agostino S, Lanzillotta D, Varano M, Botta C, Baldrini A, Bilotta A, Scalise S, Dattilo V, Amato R, Gaudio E, Paduano F, Palmieri C, Iuliano R, Perrotti N, Indiveri C, Fusco A, Gaspari M, and Trapasso F. 2018. The receptor protein tyrosine phosphatase PTPRJ negatively modulates the CD98hc oncoprotein in lung cancer cells. *Oncotarget* 9:23334-23348. 10.18632/oncotarget.25101

Ding B, Lou W, Xu L, Li R, and Fan W. 2019. Analysis the prognostic values of solute carrier (SLC) family 39 genes in gastric cancer. *Am J Transl Res* 11:486-498.

Elsnerova K, Bartakova A, Tihlarik J, Bouda J, Rob L, Skapa P, Hruda M, Gut I, Mohelnikova-Duchonova B, Soucek P, and Vaclavikova R. 2017. Gene Expression Profiling Reveals Novel Candidate Markers of Ovarian Carcinoma Intraperitoneal Metastasis. *Journal of Cancer* 8:3598-3606. 10.7150/jca.20766

Fan H, Shen YX, and Yuan YF. 2014. Expression and Prognostic Roles of TRPV5 and TRPV6 in Non-Small Cell Lung Cancer after Curative Resection. *Asian Pacific Journal of Cancer Prevention* 15:2559-2563. 10.7314/Apjcp.2014.15.6.2559

Guan X, Zhong X, Men W, Gong S, Zhang L, and Han Y. 2014. Analysis of EHMT1 expression and its correlations with clinical significance in esophageal squamous cell cancer. *Mol Clin Oncol* 2:76-80. 10.3892/mco.2013.207

Hao B, Chen Z, Bi B, Yu M, Yao S, Feng Y, Yu Y, Pan L, Di D, Luo G, and Zhang X. 2018. Role of TLR4 as a prognostic factor for survival in various cancers: a meta-analysis. *Oncotarget* 9:13088-13099. 10.18632/oncotarget.24178

He HY, Xu C, Zhao ZQ, Qin XY, Xu HM, and Zhang HW. 2011. Low expression of SLC22A18 predicts poor survival outcome in patients with breast cancer after surgery. *Cancer Epidemiology* 35:279-285. 10.1016/j.canep.2010.09.006

Huang Y, Kong Y, Zhang L, He T, Zhou X, Yan Y, Zhang L, Zhou D, Lu S, Zhou J, Zhou L, Xie H, Zheng S, and Wang W. 2018. High Expression of ITGA3 Promotes Proliferation and Cell Cycle Progression and Indicates Poor Prognosis in Intrahepatic Cholangiocarcinoma. *Biomed Res Int* 2018:2352139. 10.1155/2018/2352139

Jen J, and Wang YC. 2016. Zinc finger proteins in cancer progression. *J Biomed Sci* 23:53. 10.1186/s12929-016-0269-9

Joensuu H, Wardelmann E, Sihto H, Eriksson M, Hall KS, Reichardt A, Hartmann JT, Pink D, Cameron S, Hohenberger P, Al-Batran SE, Schlemmer M, Bauer S, Nilsson B, Kallio R, Junnila J, Vehtari A, and Reichardt P. 2017. Effect of KIT and PDGFRA Mutations on Survival in Patients With Gastrointestinal Stromal Tumors Treated With Adjuvant Imatinib

An Exploratory Analysis of a Randomized Clinical Trial. *Jama Oncology* 3:602-609. 10.1001/jamaoncol.2016.5751

Kan HP, Huang YQ, Li XH, Liu DL, Chen JJ, and Shu MJ. 2016. Zinc finger protein ZBTB20 is an independent prognostic marker and promotes tumor growth of human hepatocellular carcinoma by repressing FoxO1. *Oncotarget* 7:14336-14349. DOI 10.18632/oncotarget.7425

Ko D. 2018. ZEB2 promotes cancer cell survival and angiogenesis by cooperating with transcription factor Sp1. *Cancer Research* 78. 10.1158/1538-7445.Am2018-2028

Li L, Guo LB, Wang QS, Liu XL, Zeng YY, Wen Q, Zhang SD, Kwok HF, Lin Y, and Liu JF. 2017. DAPK1 as an independent prognostic marker in liver cancer. *PeerJ* 5. ARTN e356810.7717/peerj.3568

Li N, Zheng YH, Xuan CH, Lin ZH, Piao LZ, and Liu SP. 2015. LETMI overexpression is correlated with the clinical features and survival outcome of breast cancer. *International Journal of Clinical and Experimental Pathology* 8:12893-12900.

Linhares MM, Affonso RJ, Jr., Viana Lde S, Silva SR, Denadai MV, de Toledo SR, and Matos D. 2015. Genetic and Immunohistochemical Expression of Integrins ITGAV, ITGA6, and ITGA3 As Prognostic Factor for Colorectal Cancer: Models for Global and Disease-Free Survival. *PLoS One* 10:e0144333. 10.1371/journal.pone.0144333

Salah Z, Itzhaki E, and Aqeilan RI. 2014. The ubiquitin E3 ligase ITCH enhances breast tumor progression by inhibiting the Hippo tumor suppressor pathway. *Oncotarget* 5:10886-10900. 10.18632/oncotarget.2540

Shea LK, Honjo K, Redden DT, Tabengwa E, Li R, Li FJ, Shakhmatov M, Chiorazzi N, and Davis RS. 2019. Fc receptor-like 2 (FCRL2) is a novel marker of low-risk CLL and refines prognostication based on IGHV mutation status. *Blood Cancer Journal* 9. ARTN 47.10.1038/s41408-019-0207-7

Shen S, Wang G, Zhang R, Zhao Y, Yu H, Wei Y, and Chen F. 2019. Development and validation of an immune gene-set based Prognostic signature in ovarian cancer. *EBioMedicine* 40:318-326. 10.1016/j.ebiom.2018.12.054

Wang JW, Wei XL, Dou XW, Huang WH, Du CW, and Zhang GJ. 2018. The association between Notch4 expression, and clinicopathological characteristics and clinical outcomes in patients with breast cancer. *Oncology Letters* 15:8749-8755. 10.3892/ol.2018.8442

Yoshihara K, Tsunoda T, Shigemizu D, Fujiwara H, Hatae M, Fujiwara H, Masuzaki H, Katabuchi H, Kawakami Y, Okamoto A, Nogawa T, Matsumura N, Udagawa Y, Saito T, Itamochi H, Takano M, Miyagi E, Sudo T, Ushijima K, Iwase H, Seki H, Terao Y, Enomoto T, Mikami M, Akazawa K, Tsuda H, Moriya T, Tajima A, Inoue I, Tanaka K, and Canc JSO. 2012. High-Risk Ovarian Cancer Based on 126-Gene Expression Signature Is Uniquely Characterized by Downregulation of Antigen Presentation Pathway. *Clinical Cancer Research* 18:1374-1385. 10.1158/1078-0432.Ccr-11-2725

Zhang J, Zheng ZQ, Zheng JL, Xie T, Tian YH, Li R, Wang BY, Lin J, Xu AA, Huang XT, and Yuan YW. 2019. Epigenetic-Mediated Downregulation of Zinc Finger Protein 671 (ZNF671) Predicts Poor Prognosis in Multiple Solid Tumors. *Frontiers in Oncology* 9. ARTN 342.10.3389/fonc.2019.00342

Zhou H, Zhang Y, Wu L, Xie W, Li L, Yuan Y, Chen Y, Lin Y, and He X. 2018. Elevated transgelin/TNS1 expression is a potential biomarker in human colorectal cancer. *Oncotarget* 9:1107-1113. 10.18632/oncotarget.23275
